# Analysis of conductive olfactory dysfunction using computational fluid dynamics

**DOI:** 10.1371/journal.pone.0262579

**Published:** 2022-01-12

**Authors:** Youji Asama, Akiko Furutani, Masato Fujioka, Hiroyuki Ozawa, Satoshi Takei, Shigenobu Shibata, Kaoru Ogawa

**Affiliations:** 1 Asama Institute, Asama-ENT-Clinic, Koga, Ibaraki, Japan; 2 Department of Otolaryngology, Head and Neck Surgery School of Medicine, Keio University, Shinjuku, Tokyo, Japan; 3 Research Organization for Nano and Life Innovation, Waseda University, Shinjyuku-ku, Tokyo, Japan; 4 Department of Otorhinolaryngology, Saitama City Hospital, Saitama, Japan; Sapienza University of Rome, ITALY

## Abstract

Conductive olfactory dysfunction (COD) is caused by an obstruction in the nasal cavity and is characterized by changeable olfaction. COD can occur even when the olfactory cleft is anatomically normal, and therefore, the cause in these cases remains unclear. Herein, we used computational fluid dynamics to examine olfactory cleft airflow with a retrospective cohort study utilizing the cone beam computed tomography scan data of COD patients. By measuring nasal–nasopharynx pressure at maximum flow, we established a cut-off value at which nasal breathing can be differentiated from combined mouth breathing in COD patients. We found that increased nasal resistance led to mouth breathing and that the velocity and flow rate in the olfactory cleft at maximum flow were significantly reduced in COD patients with nasal breathing only compared to healthy olfactory subjects. In addition, we performed a detailed analysis of common morphological abnormalities associated with concha bullosa. Our study provides novel insights into the causes of COD, and therefore, it has important implications for surgical planning of COD, sleep apnea research, assessment of adenoid hyperplasia in children, and sports respiratory physiology.

## Introduction

Conductive olfactory dysfunction (COD) is a broad classification for defective olfaction resulting from the physical obstruction of airflow to the olfactory epithelium in the olfactory cleft [1]. Nasal obstruction can occur due to various causes, including nasal valve stenosis, sinusitis, and polyps [2, 3]. Endoscopic nasal surgery has shown promise in the treatment of nasal obstruction and associated disorders including rhinogenic headache [4]. However, nasal surgery should respect particular principles, based mainly on minimally invasive criteria, especially on turbinoplasty, avoiding damaging the olfactory areas [5]. COD has been associated with obstructive sleep apnea syndrome in a recent metanalysis, demonstrating a linear correlation at metaregression of pooled data included [6]. And COD has indirectly been attributed to specific causes based on questionnaire surveys and image examinations of the nasal cavity [7, 8], there is limited evidence for the obstruction to air flow in patients with COD [9]. Viral infection could develop also smell impairment acting onsite-specific for the olfactory cell as SARS-CoV-2 disease, leading to a mild nasal inflammation through a transient neurotoxic effect [10]. However the relationship between the each patient’s nasal structure and olfaction still remains to be investigated.

Leopold and subsequent research groups [11–13] noted differences in the degree of olfactory involvement between artificially configured compartments within the nasal cavity. However, they were unable to reflect the effects of distant structures. Zhao et al [14, 15] prepared anatomically accurate 3-D numerical nasal models from 0.39 mm slice CT data and measured the flow rate of olfactory clefts by steady-state analysis using the finite volume method (FVM) with an unstructured grid and a boundary adaptive grid. However, qualitative analysis cannot be performed over time, and problems with image data, geometry, and grid reproducibility remain.

In recent years, computational fluid dynamics (CFD)—a simulation method for analyzing air flow using the numerical solution of partial differential equations—has been used to study airflow within the nasal passages in both healthy and pathological settings, as well as for pre- or post-operative evaluations [16, 17]. Nevertheless, there has been minimal research specifically examining airflow within the olfactory cleft, and there are several concerns regarding the application of conventional CFD analysis in this context [18]. First, in segmentation, the measurement error increases as the slice width increases [19]. Second, mesh fidelity cannot exceed the accuracy of the original Digital Imaging and Communications in Medicine (DICOM) file. Additionally, Manmadhachary and colleagues [20] found that conversion of the file to the Stereolithography (STL) format can cause errors of up to 0.4% in bone. Third, steady state flow calculations cannot be used to accurately model changes in airflow over time [21].

In this study, we addressed these concerns by manually constructing a 3-D Cartesian grid while referencing the DICOM image at maximum resolution (0.25 mm) and calculating unsteady-state flow using direct numerical simulation (DNS). As a result, we were able to capture airflow over time within the olfactory clefts in COD patients. Next, we accounted for the presence or absence of mouth breathing in order to measure the velocity and rate of airflow in the olfactory cleft. For over half a century, researchers and clinicians have been trying to elucidate the pathophysiology of nasal obstruction that leads to mouth breathing [22]. Here, we incorporated mouth breathing into our analyses by combining the calculation of nasal cavity resistance as nasal–nasopharynx pressure drop at maximum flow (nnPD) with monitoring of carbon dioxide (CO_2_) in the air exhaled from the mouth.

To divide the COD patients into subgroups, we used a cut-off value for nnPD between combined mouth breathing (CMB) and nasal breathing (NB) Nevertheless, we acknowledged the risk of inaccurate boundary conditions because the nasal flow rate naturally decreases as the oral flow rate increases. Indeed, humans begin shifting to mouth breathing as resistance within the nasal cavity gradually increases and completely switch to mouth breathing only when the nasal cavity is completely obstructed [23, 24]. We calculated the average velocity and flow rate within the olfactory cleft of healthy control participants and compared our findings with the dynamics of olfactory cleft airflow in COD patients.

## Materials and methods

### Study design

#### Cut-off value experiment

This cross-sectional study included patients who had undergone CBCT between September 2019 and December 2019 at the Asama ENT Clinic for symptoms, including headaches, nasal congestion, or nasal discomfort associated with rhinitis (n = 232). We also included CBCT data for 9 healthy volunteers from the Asama ENT Clinic. Exclusion criteria included acute inflammation (n = 56), acute nasal discharge (n = 51), heart disease (n = 1), pulmonary disease (n = 4), individuals who were non-ambulatory (n = 1), obstructive sleep apnea syndrome (n = 15), and night time nasal obstruction (n = 9). The final sample size was of 95 patients (mean age ± standard deviation [SD]: 46.47 ± 19.3 years; 37 male, 58 female). CFD was performed on a sample of 95 people in the cut-off value experiment (**S1A Fig**). In this experiment, the power test was 0.9999 when the effect size was 1 and the alpha error was 0.01. Therefore, we conclude that the sample size in this experiment is reasonable [25].

#### COD experiment

This retrospective cohort study included participants from December 2017 to November 2019 at the Asama ENT Clinic. Two groups were included: a healthy volunteer control group (n = 9; 43.0 ± 11.6 years; 4 male, 5 female) and a COD patient group (n = 64; 46.0 ± 17.6 years; 29 male, 35 female). The control group consisted of volunteers who had no noteworthy medical history and were not taking any medications; the volunteers were individuals who visited the clinic for non-olfactory symptoms and provided consent to participate in the present study. The COD patient group had visited our specialized outpatient for olfactory disturbance, had no history of head trauma, nasal surgery, or post-viral anosmia, and showed improvements in olfactory dysfunction after functional endoscopic sinus surgery (FESS) [26]. We performed olfactory tests twice before and after surgery. These included an alinamin vein test, a standard olfactory test, and an open essence test. Patients with standard olfactory tests and/or open-essence tests judged to be non-normal were diagnosed with conductive olfactory dysfunction. In the control group, the results of all three tests were found to be normal.

Previous studies have reported nasal polyps in the lower and middle nasal passages and olfactory cleft as causes of olfactory dysfunction [27]. Although nasal polyps are physical obstacles to airflow that can be clearly observed during physical examinations, there is a high degree of variability in polyp shape between patients and polyps may be exacerbated by other morphological abnormalities, such as septal deviation. Accordingly, we classified the COD patient group as patients with COD without nasal polyps (CODsNP) (n = 31; 45.2 ± 18.5 years; 13 male, 18 female) and patients with COD having nasal polyps (CODwNP) (n = 33; 46.7± 16.7 years; 16 male, 17 female). Nasal polyps were identified using CBCT and nasal endoscopy, and CODwNP patients were excluded, as previously discussed. Using the cut-off value established in the cut-off value experiment, the CODsNP patients below a cut-off value were defined as having nasal breathing (NB; CODsNP_NB) (n = 11; 52.9 ± 14.1 years; 6 male, 5 female) and those above a cut-off value were defined as having combined oral breathing (CMB; CODsNP_CMB) (n = 20; 41.0 ± 19.2 years; 7 male, 13 female). (**S1B Fig**). In this experiment, the power test for sample size was 0.9865 when the effect size was set to 1 and the alpha error was 0.01. Therefore, we conclude that the sample size in this experiment is reasonable [25].

### Ethical considerations

This study was conducted according to the tenets of the Declaration of Helsinki and with approval from the Ethical Review Board of the Ibaraki Seinan Medical Center Hospital (Approval No. 30–1–22). Participants provided written informed consent following a verbal explanation of the study design, including purpose, survey details, and protection of personal information. All data collected for this study were deidentified using participant codes. Moreover, the data were analyzed as a group and only used for the research purposes described prior to obtaining written informed consent.

### CBCT imaging

A three-dimensional model was created for each participant from raw movie data captured using CBCT (3D Accuitomo F17, J. Morita, Kyoto, Japan). The geometry of the nasal cavity was acquired from the CBCT scans (voxel size = 0.25 mm, slice thickness = 0.25 mm) as DICOM slices using semi-automatic mesh generator software (iCFD, Tokyo, Japan). CBCT has lower radiation exposure and finer resolution than conventional CT. To prevent confounding effects of the nasal cycle [19], CBCT was performed immediately after nasal treatment with Bosmin Xylocaine gauze.

### Mesh model generation

We used our own mesh generator software (iCFD, Tokyo, Japan) to generate structured grids by checking each box with a side of 0.25 mm against the same part of DICOM geometric data. We adopted a 3-D Cartesian grid because pressure resistance is more important than wall friction resistance. It is preferable to arrange the grid points in an almost laminar flow so as to resolve the entire flow path without collecting the grid near the wall, and the regularity of the grid point arrangement is important to reduce the calculation time. Although there are individual differences, for example, the number of grids is about 15 million = 160 (transverse 4 cm) x 400 (sagittal 10 cm) x 240 (vertical 6 cm), and about one sixth of that when considering only the nasal cavity.

### Finding a nnPD cut-off value

We plotted a receiver operated characteristic (ROC) curve of sensitivity versus 1 –specificity for all possible cut-off values between cases (those included in the cut-off value experiment) and controls. We determined a cut-off value for mouth breathing, defined as the optimal cut-point value minimizing the summation of absolute values of the differences between the area under the curve for sensitivity and the area under the curve for specificity at which the difference between sensitivity and specificity was at a minimum.

### CFD analyses

All CFD models were created using Nagare (iCFD, Tokyo, Japan). We calculated the unsteady-state of the almost laminar flow between the nostrils and epipharynx using DNS, without approximate calculation models, (time for inspiration = 2.5 s, time for exhalation = 2.5 s, flow rate [tidal volume] = 630 mL, non-slip wall condition). The flow rate mimics the human respiratory curve using a sine curve. The site of inspiratory outflow is the lower nasopharynx, while the nostrils are the sites of expiratory outflow. We visualized and calculated the pressure drop, velocity, and flow rate within the olfactory cleft bilaterally in the same interval used for cut-off point examination using ParaView (Los Alamos National Laboratory et al, New Mexico, USA). The pressure drop—indicating energy dissipation within the measurement interval—was defined as the pressure difference between the plane containing nasal columella and the lower plane of the epipharynx at maximum flow, or nnPD.

### Mouth exhalation CO_2_ detection test

A recordable CO_2_ monitor (WEC-7301, Nihon Kohden, Tokyo, Japan) was used for the mouth exhalation CO_2_ detection test. We made a measurement mask by modifying a medical oxygen mask, cutting the outer edge and molding it to fit the curves of the face using a heat gun. The aluminum plate in the upper part of the modified mask fit closely to the upper lip without a gap, allowing the monitor to measure only the CO_2_ exhaled from the mouth. We assessed mouth breathing in each participant by measuring CO_2_ exhaled from the mouth for 10 minutes in a seated position. This test was performed in a well-ventilated, humidity-controlled room with white walls and normal LED lighting (ambient temperature = 25°C, humidity = 50%).

### Statistical analysis

Date are expressed as means ± standard error of measure (SEM). Statistical analysis was performed using the GraphPad Prism version 8.2.1(441) (GraphPad Software, San Diego, CA; https://www.graphpad.com/scientific-software/prism/). We assessed that data for normal vs non-normal distribution and equal vs biased variation using the D’Agostino-Pearson test/Kolmogorov-Smirnov test and F value test/Bartlett’s test, respectively. Parametric analysis was conducted using a Student’s t-test or one-way ANOVA with a Tukey test (if the interaction was significant) or Sidak test (if the interaction was not significant but the main effect was significant) for post-hoc analysis.

## Results

### Cut-off value for nnPD

**Fig 1:**
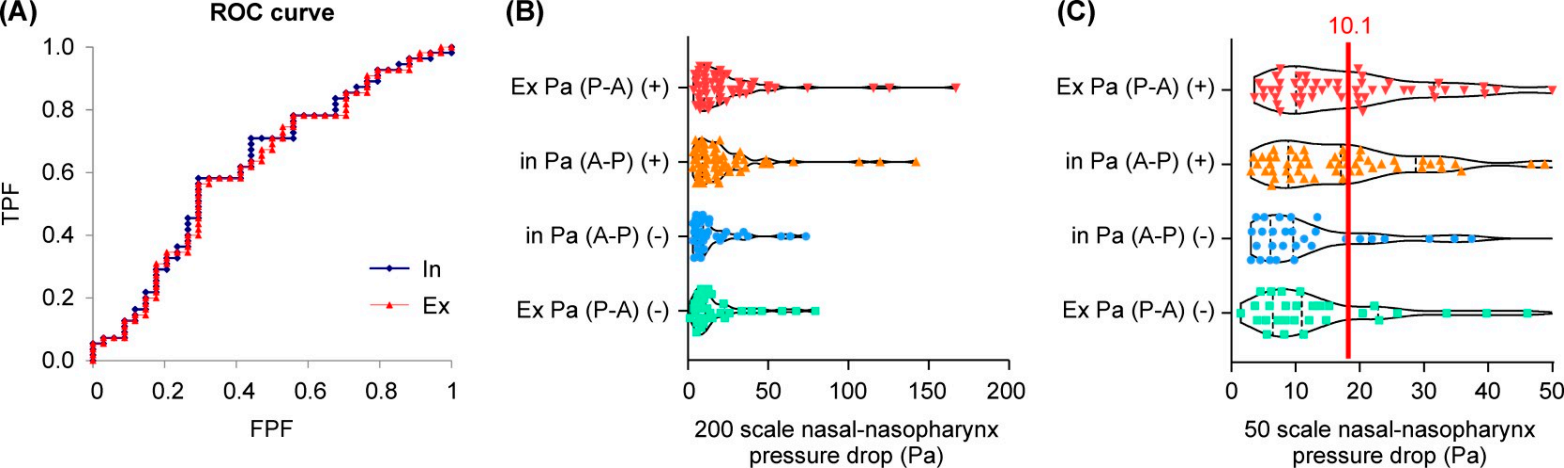
Receiver operating characteristic (ROC) curve and Nasal-nasopharyngeal pressure drop at maximum flow (nnPD). **a.** Receiver operated characteristic (ROC) curve in patients with mouth breathing and nasal breathing in exhalation and inspiration. The cut-off point was taken as the point closest to the upper left corner of the ROC curve. **b. c.** Nasal-nasopharynx pressure drop at maximum flow (nnPD) values during inhalation (In) and exhalation (Ex). **b.** nnPD values for participants with (+) and without (-) mouth breathing plotted on a scale from 0–200 Pa. **c.** nnPD values for participants with (+) and without (-) mouth breathing plotted on a scale from 0–50 Pa. The cut-off point calculated from the ROC curve was indicated by the red line.

We obtained the nnPD results and calculated the cutoff value. Results of the cut-off value experiment revealed that the CMB group had significantly higher nnPD compared to the NB group (F_(3966,71.38)_ = 7.94, *p* < 0.05). There was no significant difference between left and right sides (F = 4.92, p = 0.18) or between expiration and inspiration (F = 4.98, *p* = 0.18). The results of the mouth breathing test were plotted as an ROC curve. The cut-off value corresponds to the point closest to the upper left corner of the ROC curve (Fig 1A). Both expiration and inspiration were 10.01 Pa, and there was no significant difference between the left and right sides (F = 4.96, p = 2.01) (Fig 1B and 1C). We were able to determine the cut-off value for mouth breathing, which was used to subdivide patients into two groups in the COD experiment. In addition, none of the demographic indicators showed a correlation by gender, age, or body mass index. (S1A and S1B Table).

The cutoff value was not high. However, we investigated the area under the ROC curve (AUC). The AUC of IN was 0.62 (p <0.05), and the AUC of EX was 0.62 (p <0.05), both relatively low, but statistically significant. The True Positive Fraction was 0.58 for IN and 0.56 for EX, while the False Positive Fraction was 0.29 for IN and 0.29 for EX. Thus, despite their significance, we need to interpret the AUC results carefully.

### Velocity in the olfactory cleft

In the COD experiment, we determined the airflow velocity in the olfactory cleft at maximum flow in two types of CODsNP patients, classified based on the cut-off value established in the cut-off value experiment: CODsNP_NB patients, defined by an nnPD value below the cut-off value; and CODsNP_CMB patients, defined by an nnPD value above the cut-off value.

**Fig 2:**
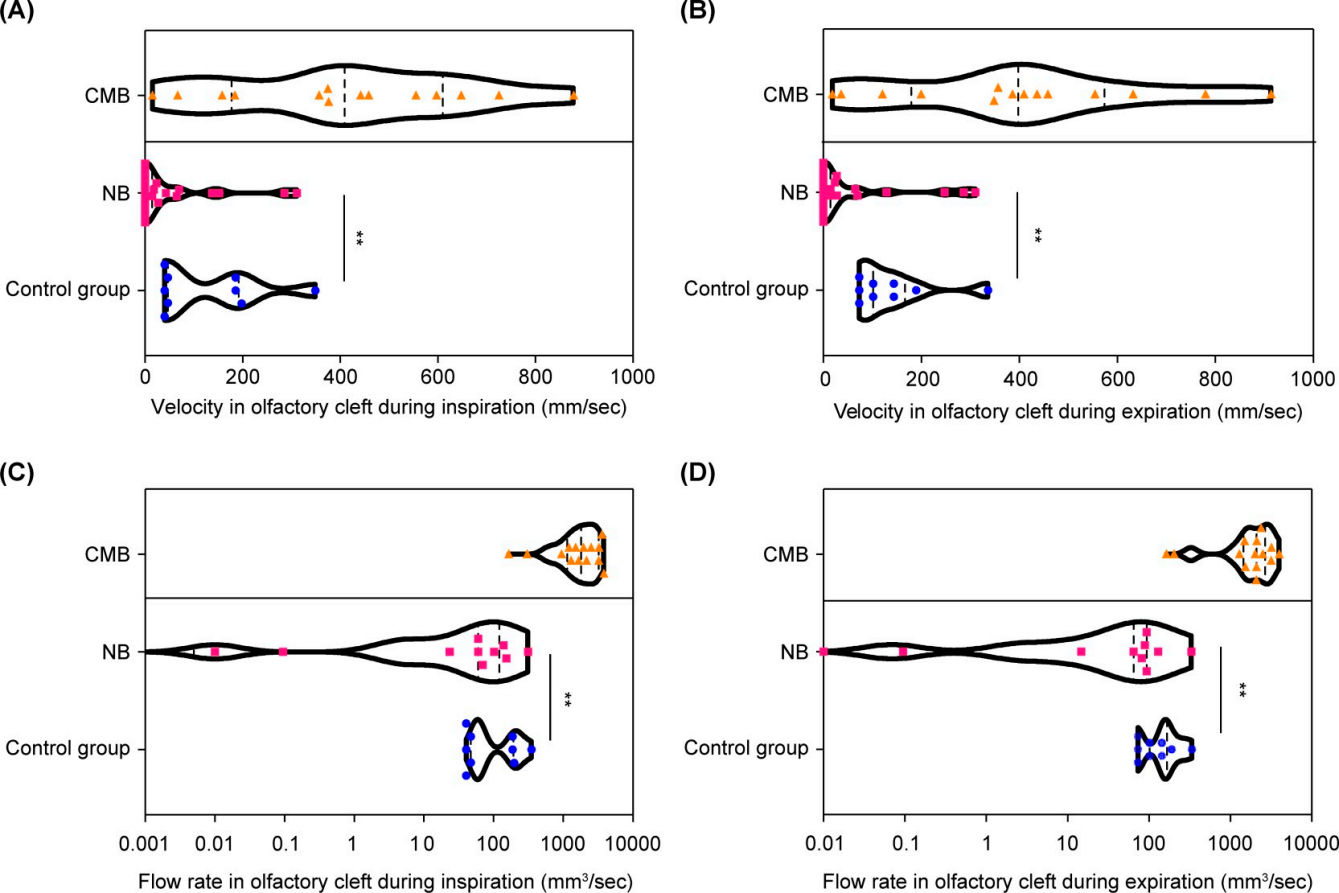
Velocity and flow rate in the olfactory cleft during inspiration and expiration for the combined mouth breathing (CMB), nasal breathing (NB), and control groups. **a**. The velocity in the olfactory cleft at maximum flow during inspiration. **b**. The velocity in the olfactory cleft at maximum flow during expiration. **c**. The flow rate in the olfactory cleft at maximum flow during inspiration. **d**. The flow rate in the olfactory cleft at maximum flow during expiration. The CMB group is presented as reference values. Comparison between the NB and control groups revealed a significant difference: ** p < 0.01.

We then compared the olfactory dysfunction group with the nasal breathing and control groups. Inspiratory velocity was significantly lower in the CODsNP_NB group compared to the healthy control group (F_(18,54)_ = 2.1, *p* < 0.01) (Fig 2A). Nearly half (49.87%) the subjects in the CODsNP_NB group had a velocity value of 0 mm/sec. Expiratory velocity was also significantly lower in the CODsNP_NB group as compared to that in the control groups (F_(18,54)_ = 2.1, *p* < 0.01) (Fig 2B). 51.5% of individuals in the CODsNP_NB group had a velocity value of 0 mm/sec. There was no significant difference between expiration and inspiration velocity (F = 4.70, *p* = 0.71), and no significant difference in velocity between left and right sides (F = 4.89, *p* = 0.16). For the control group, the mean velocity in olfactory cleft at maximum flow was 192.7 mm/s during inspiration and 206.1 mm/s during expiration (Fig 2A and 2B).

### Flow rate in the olfactory cleft

We also determined the airflow rate in the olfactory cleft at maximum flow in the two types of CODsNP patients. Inspiratory flow rate in the CODsNP_NB group was significantly higher than in the control group (F_(18,54)_ = 3.95, *p* < 0.01) (Fig 2C and 2D). There were no significant differences between expiration and inspiration flow rate (F = 4.91, *p* = 2.00), and no significant differences in flow rate between left and right sides (F = 4.88, *p* = 0.16). For the control group, the mean flow rate in olfactory cleft at maximum flow was 126.1 mm^3^/s during inspiration and 137.6 mm^3^/s during expiration (Fig 2C and 2D).

### Morphological evaluation of cone beam computed tomography (CBCT) scan data and a pattern diagram

**Fig 3:**
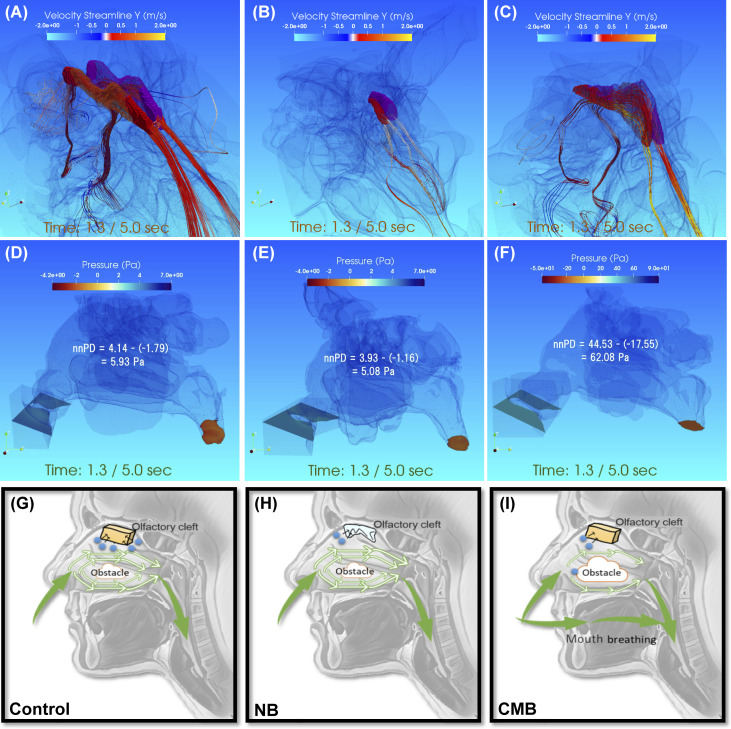
Characterization of a typical participant and conceptual diagram. **a.**-**c.** Characterization of a typical participant in the conductive olfactory disorder without nasal polyps (CODsNP). The olfactory cleft is colored in red (right side) and purple (left side). Streamline diagram under maximum pressure during inspiration from a right anterolateral view. **a.** Control: bilateral olfactory clefts have a normal shape. Air flows from the front of the olfactory cleft to the back during inspiration. **b.** Nasal breathing (NB): the latter half of the bilateral olfactory clefts does not exist. The flow velocity is slower than that of the control. **c.** The combined mouth breathing (CMB): bilateral olfactory clefts have a nearly normal structure, but the slit is thinner and slightly curved compared to the control. The flow velocity is faster than that of the control. **d.**-**f.** Pressure field at the plane containing nasal columella and the lower nasopharynx, and nasal-nasopharynx Pressure Drop (nnPD) during inspiration. **d.** Control: nnPD is 5.93 Pa. **e.** Nasal breathing (NB): nnPD is 5.08 Pa. **f.** Combined mouth breathing (CMB): nnPD is 62.08 Pa. There is one more digit on the scale in Figures d and e. **g.-i.** We created pattern diagrams based on the scanned data of the Control group, CODsNP_NB patients, and CODsNP_CMB patients. The “obstacle” represents the airflow difficulties through the lower turbinate, the middle turbinate, the upper turbinate, the nasal septum, and the nasopharynx. The blue dots represent an odorous substance. A slit without stenosis of the olfactory cleft is represented by an orange block and an olfactory cleft with structural abnormalities, such as stenosis and blind ends, is represented by a light blue polygon. The green arrows show the direction of airflow during inspiration.

We performed morphological evaluation using the CBCT scan (Fig 3A–3C). And based on the scanned data, pattern diagrams of the Control group, CODsNP_NB patients, and CODsNP_CMB patients were created (Fig 3D–3F).

**Fig 4:**
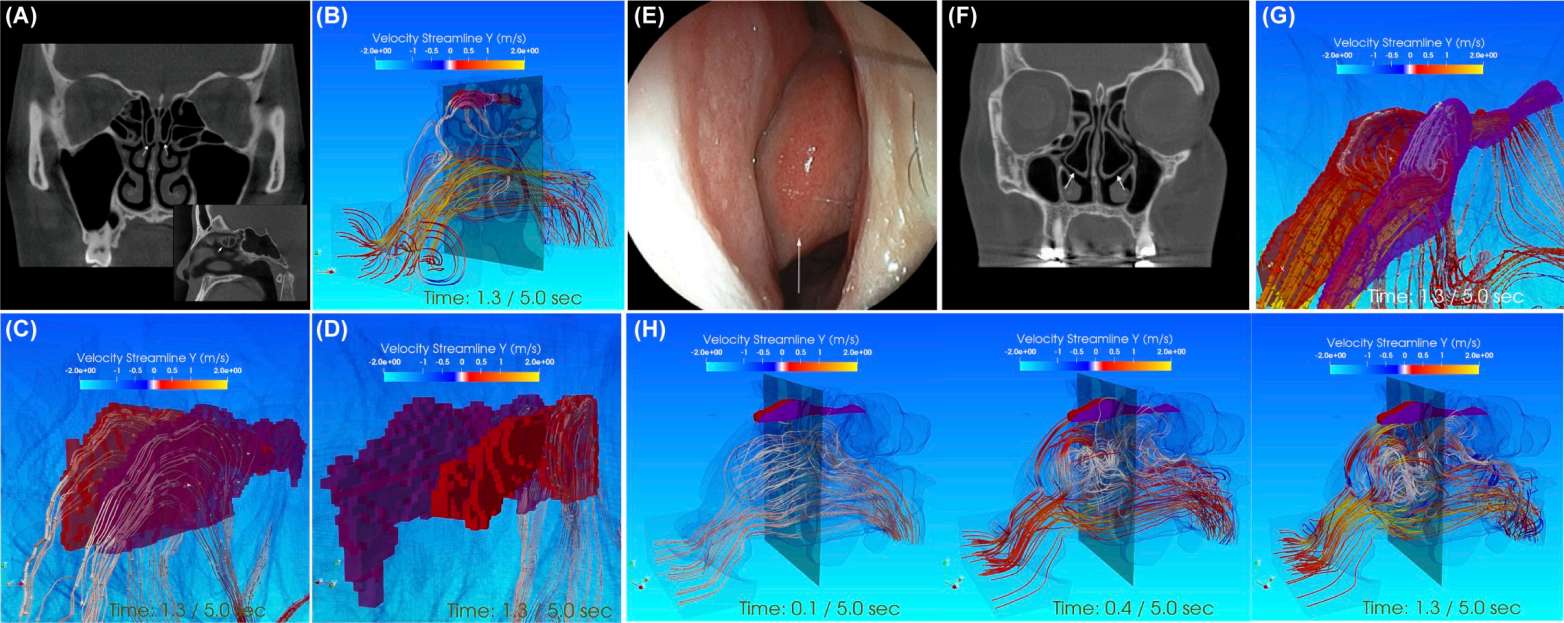
Two cases already known to cause COD. **a.-g.** Two cases already known to cause COD. **a.-d.** Characterization of the pneumatized superior turbinates in a participant in the conductive olfactory disorder without nasal polyps (CODsNP) group. **a**. Cone beam computed tomography (CBCT) scan in the coronal plane showing bilateral pneumatized superior turbinates (arrows). The sagittal plane showing a pneumatized superior turbinate on the left side (arrow). **b**.**–d**. The olfactory cleft is colored in red (right side) and purple (left side). **b**. Streamline diagram under maximum pressure during inspiration from a left anterolateral view. The velocity of airflow from the nasal vestibule toward the dorsal aspect of the olfactory cleft increases from red to yellow. **c**.**, d**. Streamline diagram under maximum pressure during inspiration around the olfactory cleft. The dorsal half of the bilateral olfactory clefts are tubular and blind ended. White indicates a relatively slow airflow velocity. The calculation grid is a cube with a slide length of 0.25 mm. **c**. Left anterolateral view. **d**. Right posterolateral view. **e.-h.** Characterization of concha bullosa in a participant in the conductive olfactory disorder without nasal polyps (CODsNP) group. **e**. Nasal endoscopy images of the left concha bullosa (arrow). **f**. Cone beam computed tomography (CBCT) scan in the coronal plane showing bilateral pneumatized middle turbinates (arrows). **g**.**, h**. The olfactory cleft is colored in red (right side) and purple (left side). **g**. Streamline diagram under maximum pressure during inspiration around the olfactory cleft. The bilateral olfactory clefts have a normal shape. The velocity of the bilateral olfactory cleft at the time of 1.3 s is over 2000 mm/s, and it does not actually occur because of combined mouth oral breathing. **h**. Streamline animation changing with inspiration time viewed from the left front (upper picture series). The olfactory cleft is colored: right (red cubes), left (purple cubes). In our simulation, a relatively slow speed vortex occurred in front of the bilateral middle turbinates, 0.1–0.4 s after the start of inspiration. It becomes the maximum pressure gradient at the time of intake after 1.3 s have passed. E.g., The mucous membrane was red in the part of the middle turbinate where the slow velocity vortex was occurring.

Morphological evaluation using CBCT scans revealed that CODsNP_NB patients had an abnormal olfactory cleft shape, stenotic slit, and/or lumen structure (Figs 3B, 3E, 3H; 4A–4D), while CODsNP_CMB patients typically had an anatomically normal olfactory cleft structure (Figs 3C, 3F, 3I; 4E–4H). A control group has also been compared to the patients. (Fig 3A, 3D and 3G).

### Examples of detailed analysis according to clinical condition

In the COD experiment, we encountered two cases with common morphological abnormalities in the nasal cavity. The first case was of a pneumatized middle turbinate (concha bullosa). Since the wall of the middle turbinate is continuous with the olfactory cleft, concha bullosa tends to cause stenosis of the olfactory cleft or inhibit the inflow of air (Fig 4C–4H) [28, 29]. The second case was of a pneumatized superior turbinate, extending anteriorly between the middle turbinate and the septum leading to anosmia or hyposmia due to obstruction of the olfactory cleft (Fig 4A–4D) [30].

### Relationship of nnPD between inspiration and expiration

When comparing the nnPD value between inspiration and expiration, many participants had an odds ratio (inspiration/expiration) less than 1.0: 96/100 participants in the cut-off point experiment, and 25/31 participants in the COD experiment (CODsNP group).

## Discussion

In this study, we established the cut-off value at which nnPD suggests the presence of mouth breathing and used this for CFD analyses [19]. According to the Navier–Stokes equation, nnPD approaches infinity when the flow pathway is blocked, which results in nnPD values that cannot be physiologically attained *in vivo*. Instead, humans increase breathing effort by switching to mouth breathing once the nnPD exceeds this physiological threshold.

As described in the COD experiment results, the CODsNP_NB group showed significantly lower velocity and flow rate within the olfactory cleft than the healthy control group. This is likely due to the shape of the olfactory cleft: a circular tube structure or partial stenosis of the olfactory cleft slit due to mucosal adhesion resulted in a reduced velocity and flow rate. While routine nasal screening tests using CBCT often reveal abnormalities in the shape of the olfactory cleft, these findings do not necessarily confirm the presence of CODsNP.

Despite using different calculation methods than those used in this study, a previous study on sleep apnea patients also reported that airflow was smoother with NB than with CMB [31]. Considering that excessively high nnPD values are physiologically unattainable *in vivo*, the corrected velocity and flow rate within the olfactory cleft that was recalculated using reduced tidal volume, can be considered the nnPD value for CODsNP_CMB. However, if we assume that the concentration of odorous substances in the olfactory cleft is equivalent to the concentration near the nasal inlets (nostrils), there is a contradiction in the mechanism of how COD occurs in CODsNP_CMB patients. In order to address this contradiction, we hypothesize that the concentration of odorous substances in the olfactory cleft is greatly reduced when there is a physical narrowing of the nasal passages. It is widely accepted that the nasal mucosa functions as an air filter; therefore, it is reasonable to assume that the concentration of odorous substances may be greatly reduced in obstructed pathways from the nostrils to the olfactory cleft (Figs 3C, 3F; 4E and 4H). In addition, the residence time of substances in the olfactory region is likely an important factor for olfactory function and one that may be affected by narrower or obstructed airways.

In terms of the relationship of nnPD between inspiration and expiration, we found that the nnPD was smaller for inspiration than for expiration in many subjects. In the nasal cavity—a bidirectional flow path—the nasal resistance of inspiration is smaller than that of expiration. This relationship is also seen on the general healthy human breathing curve [32] that illustrates greater pressure and shorter duration for inspiration than for expiration. This validates the notion in sports medicine that runners should inhale through the nose and exhale through the mouth to reduce airway resistance and improve efficiency of oxygen transport.

Two examples we investigated, which have already been given anatomical names and suggested to have olfactory disturbance, but few studies have investigated the causal relationship between structural abnormalities and olfactory disorders (Fig 4A–4H). Therefore, an unknown COD disorder may be newly found by our research. And more, we expanded the olfactory cleft to the highest precision, and performed time-dependent dynamic analysis and spatial structure-dependent flow analysis. We obtained the additional information of visually understanding the channels that are beneficial for treatment. Additional information can also be targeted during surgical treatment, which has the benefit of minimizing the extent of the invasion.

Several limitations of this study should be acknowledged. First, the cut-off value established in this study was used as one guideline for dividing NB and CMB, but it is not an absolute parameter. Because in daily medical care we encounter some patients with sleep apnea syndrome or a history of adenoidectomy in childhood had habitual mouth breathing despite nnPD below the cut-off value. Next, since the subjects of this study are only Asian Japanese, the cut-off value (10.01 Pa) may not be suitable for other races. In the cutoff experiment, patients with sleep apnea syndrome, which has been reported to correlate with body mass index, were excluded. As far as the data of mouth breathing and nasal breathing that have already been reported, there is no correlation with body mass index [33]. However, it is necessary to investigate the correlation with body mass index using the measurement method used in this study in the future.

For the realistic analysis of combined nasal-oral breathing, it is necessary to widen the CBCT imaging range with the mouth completely open and to reset the entrance and exit boundary conditions. This would be an important area of focus for future research.

Despite the primitive nature of our fluid analysis method, its simplicity makes it highly reproducible and easy to understand. Although it may be possible in the future to improve the grid design to reproduce the sophisticated geometry of the nasal cavity and to predict detailed air flow characteristics, especially near the wall, we believe that for nasal CFD, our method simplifies the calculation, optimizes the grid, and promotes the fusion of medicine and engineering.

## Conclusions

We established the cut-off value of nnPD (10.01 Pa) that corresponds with the onset of mouth breathing. We found that decreased velocity and flow rate in the olfactory cleft underlies olfactory disturbance in COD. Furthermore, we established average values for the olfactory cleft velocity and flow rate in healthy olfactory subjects and obtained the target surgical values for COD patients. Finally, even if the shape of the olfactory cleft was anatomically normal, we found that COD may be caused by a decrease in the concentration of odorous substances due to the constriction of the flow path leading to the olfactory cleft. The results of the present study facilitate better understanding of nasal structures that can be potential involved in COD. Highlights how the results of our study could still be limited and need further scientific evidence. However this new information may help in the planning of more effective surgical intervention for COD, clinicians maybe able to identify retrospectively identify the cause of COD by using the patient’s CBCT data related to olfactory disorders obtained in the past.

## Supporting information

S1 Fig Study design. **A.** the cut-off value experiment. **B.** the total number of CFD analyses.
(TIF)

S1 Table The demographic table describing patients’ features. **A. B.** The demographic table describing patients’ features was shown. The numbers in the table show the average value of each indicator. The numbers in parentheses indicated the standard deviation of each indicator.
(TIF)
