# Supplementary figures and images for: Analysis of conductive olfactory dysfunction using computational fluid dynamics
Source: PLoS One. 2022 Jan 12;17(1):e0262579. doi: 10.1371/journal.pone.0262579 (PMC8754295; doi:10.1371/journal.pone.0262579)

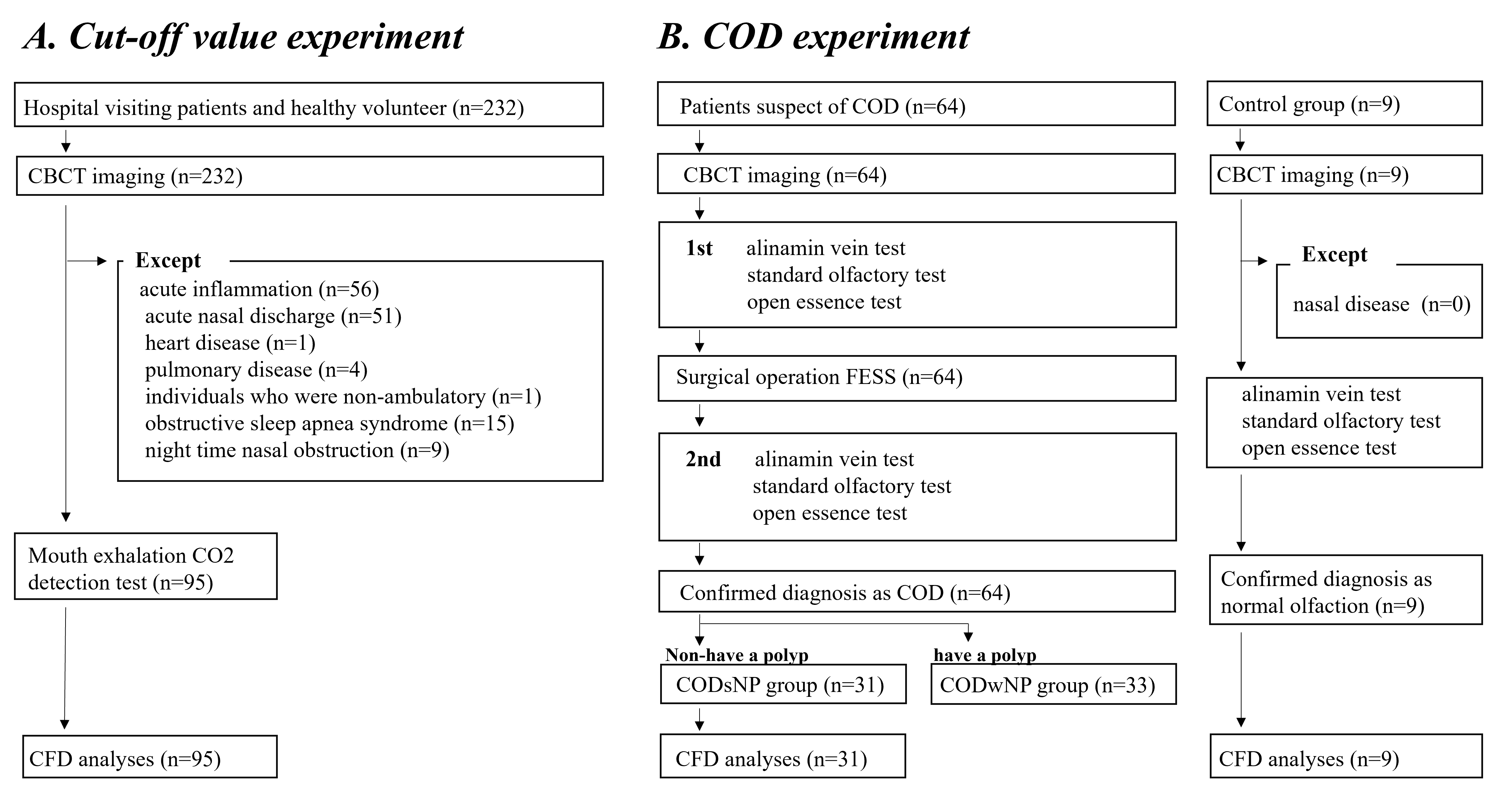

Supplement: S1 Fig — A. the cut-off value experiment. B. the total number of CFD analyses. (TIF) [file pone.0262579.s001.tif]

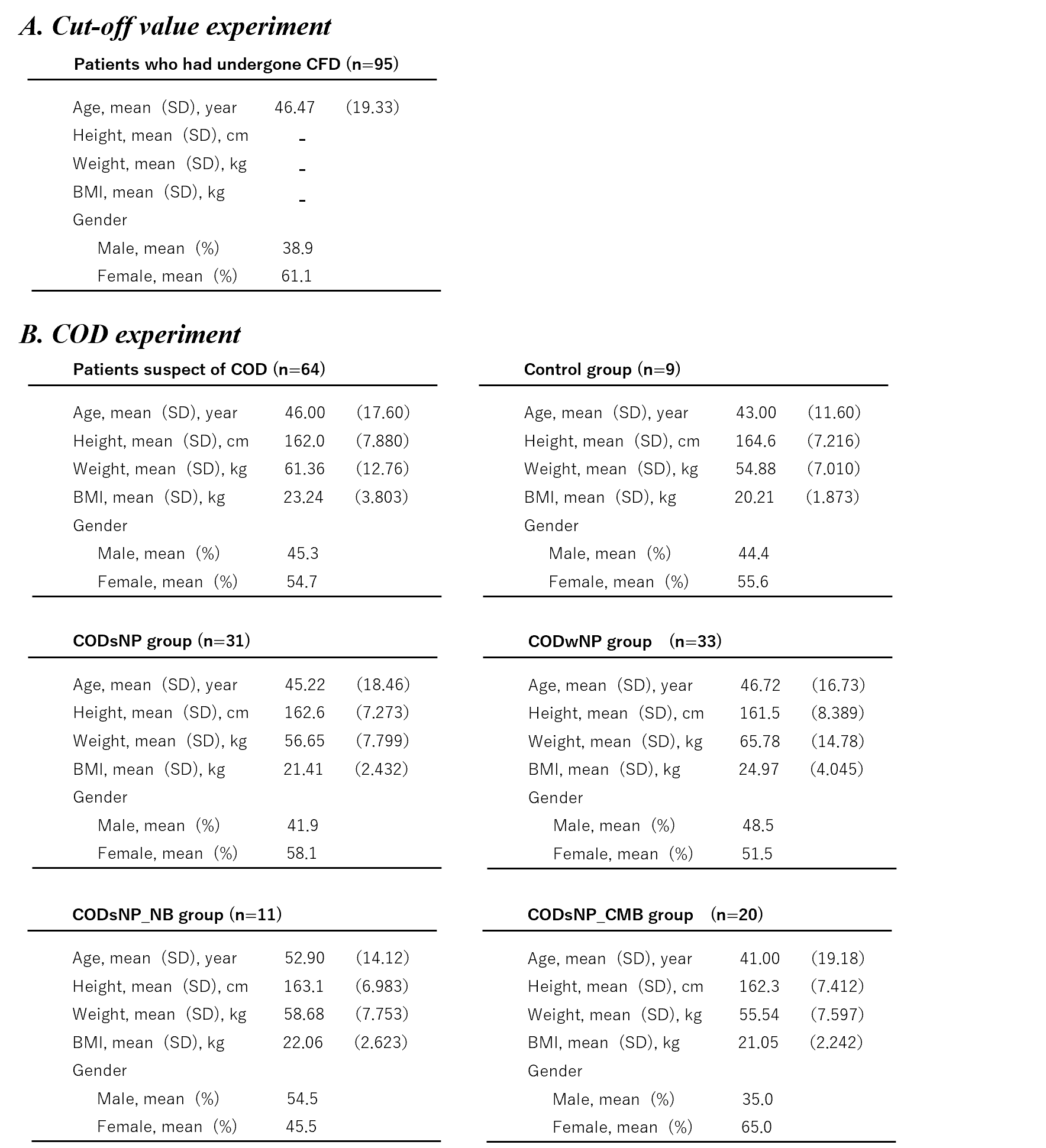

Supplement: S1 Table — A. B. The demographic table describing patients’ features was shown. The numbers in the table show the average value of each indicator. The numbers in parentheses indicated the standard deviation of each indicator. (TIF) [file pone.0262579.s002.tif]
